# Prevalence of common α-thalassemia determinants in south Brazil: Importance for the diagnosis of microcytic anemia

**DOI:** 10.1590/S1415-47572010005000086

**Published:** 2010-12-01

**Authors:** Sandrine C. Wagner, Simone M. de Castro, Tatiana P. Gonzalez, Ana P. Santin, Leticia Filippon, Carina F. Zaleski, Laura A. Azevedo, Bruna Amorin, Sidia M. Callegari-Jacques, Mara H. Hutz

**Affiliations:** 1 Departamento de Genética, Universidade Federal do Rio Grande do Sul, Porto Alegre, RSBrazil; 2 Centro Universitário FEEVALE, Novo Hamburgo, RSBrazil; 3 Faculdade de Farmácia, Universidade Federal do Rio Grande do Sul, Porto Alegre, RSBrazil

**Keywords:** alpha-thalassemia, Brazilian population, genotype, hemoglobin, microcytosis

## Abstract

Alpha thalassemia has not been systematically investigated in Brazil. In this study, 493 unrelated individuals from the southernmost Brazilian state of Rio Grande do Sul were screened for deletional forms of α-thalassemia. One hundred and one individuals had microcytic anemia (MCV < 80 fL) and a normal hemoglobin pattern (Hb A _2_ < 3.5% and Hb F < 1%). The subjects were screened for - α^3.7^ , - α^4.2^ , - α^20.5^ , — ^SEA^ and — ^MED^ deletions but only the - α^3.7^ allele was detected. The - α^3.7^ allele frequency in Brazilians of European and African ancestry was 0.02 and 0.12, respectively, whereas in individuals with microcytosis the frequency was 0.20. The prevalence of α-thalassemia was significantly higher in individuals with microcytosis than in healthy individuals (p = 0.001), regardless of their ethnic origin. There were also significant differences in the hematological parameters of individuals with - α^3.7^ / αα, - α^3.7^ /- α^3.7^ and β-thalassemia trait compared to healthy subjects. These data suggest that α-thalassemia is an important cause of microcytosis and mild anemia in Brazilians.

The thalassemias are a diverse group of microcytic hemolytic anemias characterized by defective synthesis of the α or β globin chain that results in α-thalassemia and β-thalassemia, respectively (Kazazian, 1990). The molecular lesions associated with α-thalassemia can be classified as α^+^ or α^0^ defects, depending on whether they partially or completely abolish α-globin chain production, respectively (Higgs and Weatherall, 2009).

The spectrum of mutations that underlie α-thalassemias varies considerably among different populations. This finding suggests that these mutations almost certainly arose locally and that their frequency has expanded through a combination of natural selection, founder effect and gene drift. There is strong evidence that natural selection reflects past or present exposure to malaria. Overall, the α-thalassemias follow a similar distribution to the β-thalassemias, extending from sub-Saharan Africa through the Mediterranean region and Middle East, to the Indian sub-continent and East and South-East Asia (for a review see Higgs and Weatherall, 2009). The most common α^+^-thalassemia deletional alleles are -α^3.7^ and -α^4.2^. The -α^3.7^ allele has been observed worldwide, with higher frequencies in some African and Mediterranean populations. The -α^4.2^ is most common in Asian countries although it also occurs in Mediterranean populations (Flint *et al.*, 1993). The -α^20.5^ and —^Med^ alleles are also frequent among Mediterranean populations (Kattamis *et al.*, 1996). Non-deletional α-thalassemias are generally less frequent and have limited geographical distributions when compared to the deletional forms of the disease (Higgs and Weatherall, 2009).

The current Brazilian population was formed by successive migratory waves. Amerindians already occupied Brazilian territory when the Portuguese arrived in 1500 and colonized the country. From the 16^th^ to 19^th^ centuries Africans were brought to Brazil as slaves, with other migratory waves of Europeans occurring in the 19^th^ and 20^th^ centuries, mainly from Italy, Germany and Spain (Salzano and Bortolini, 2002). All of these migratory events have contributed to the formation of a highly admixed multiethnic population. This heterogeneity has been documented in several genetic studies using uniparental or autosomal markers that revealed a typical, non-uniform triethnic (European+African+Amerindian) pattern for the Brazilian population gene pool. Southern populations generally have lower African and higher European contributions when compared to other Brazilian groups (Salzano and Bortolini, 2002; Callegari-Jacques *et al.*, 2003; Parra *et al.*, 2003; Zembrzuski *et al.*, 2006; Leite *et al.*, 2008, 2009). The migration of people mainly from sub-Saharan Africa and the Mediterranean region introduced thalassemias into the present-day Brazilian population since there is no evidence for autochthonous thalassemias in Brazilian Amerindians (Zago *et al.*, 1995).

Although several investigations have reported the prevalence of hemoglobin disorders in Brazil, α-thalassemia in particular has not been systematically investigated. Few studies have screened Brazilians to estimate the prevalence of α-thalassemia in the general population. Sonati *et al.* (1991) observed a prevalence of 21% heterozygous -α^3.7^/αα and 2% homozygous -α^3.7^/-α^3.7^ genotypes in African Brazilian blood donors from the southeast. In another survey in a highly admixed northeastern population the -α^3.7^/αα genotype was detected in 20% of 514 newborn babies while the homozygous genotype was seen in 2.5% of the children investigated (Adorno *et al.*, 2005). In a recent study, the frequency of the -α^3.7^/αα genotype in a healthy admixed northern Brazilian population was 7% (Souza *et al.*, 2009). The present study was done to determine the prevalence of α-thalassemia in southern Brazilians of African and European ancestry and in a sample of patients with mild anemia, microcytosis and normal iron status from the same population.

493 unrelated individuals were enrolled in this study. The largest sample consisted of 392 volunteers (191 African Brazilians and 201 Brazilians of European ancestry) recruited randomly during free routine laboratory blood determinations in the School of Pharmacy at the Federal University of Rio Grande do Sul (UFRGS); the subjects had been sent for these determinations by city health centers. The blood samples were collected in 2001 and 2002. The remaining 101 subjects were recruited from individuals referred to the Hemoglobin Center of the same School to investigate microcytosis with no iron deficiency, chronic condition or intestinal parasitic worms. This study was approved by the Ethics Committee at UFRGS, and all of the participants provided written informed consent prior to participating in the study.

The blood samples were collected in vacuum tubes with EDTA as anticoagulant. The hematological indices were obtained with an automated counter (ABX Micros 60, Horiba Group, Japan). For those subjects referred to the Center because of microcytosis (MCV < 80 fL), the inclusion criterion was a previous diagnosis compatible with the possible presence of α-thalassemia based on a normal Hb profile evaluated by high-performance liquid chromatography (HPLC; Bio-Rad Variant-Beta-Thal Short Program) and/or isoelectric focusing (IEF; Perkin-Elmer). All of the subjects had Hb A_2_ < 3.5% and Hb F < 1%. All of the patients had a normal iron status and/or a clinical suspicion of α-thalassemia (no response to iron therapy).

Genomic DNA was obtained from peripheral blood by a salting-out procedure (Lahiri and Nurnberger, 1991). The α-globin genotypes were screened with the polymerase chain reaction (PCR) in multiplex reactions as described by Tan *et al.* (2001) or in single reactions with the primers and protocols described by Dode *et al.* (1993). The genotypes were determined after electrophoresis of the amplicons in 1% agarose gels containing ethidium bromide. A 100 bp ladder was used to score the band sizes and a known heterozygous sample was run as a positive control in all gels.

The genotypic distribution and allele frequencies were estimated by gene counting. Deviation from Hardy-Weinberg equilibrium was tested by the chi-square test. The genotypic and allele frequencies among groups were compared using the chi-square test or, when appropriate, Fisher's exact test. All statistical comparisons were done using the WinPEPI program (Abramson, 2004) with a value of p < 0.05 indicating significance.

Hemoglobin (Hb), hematocrit (Hct) and red blood cell (RBC) values were corrected for age and sex. Multivariate Analysis of Variance (MANOVA) was used to compare these adjusted variables, as well as the mean corpuscular volume (MCV), mean corpuscular hemoglobin (MCH) and mean corpuscular hemoglobin concentration (MCHC), with the corresponding values in patients with microcytic anemia (several α-genotypes), in a previously characterized group of 124 β-thalassemia trait carriers from the same population (Reichert *et al.*, 2008) and in healthy (control) volunteers. The SNK procedure was used for subsequent pairwise multiple comparisons between groups, except for RBCs, for which Tamhanes test was used to account for heterocedasticity. These tests were done using SPSS v12.0.

The allele and genotypic frequencies for the various groups are shown in Table 1. All of the patients with microcytosis were screened for -α^3.7^, -α^4.2^, -α^20.5^, —^SEA^ and —^MED^ deletions but only the -α^3.7^ allele was detected. Since this allele was the only one observed in microcytic patients, the investigation in volunteers was also restricted to the -α^3.7^ deletion. Among 201 Euro-descendents, 9 (4.5%) were heterozygous for -α^3.7^/αα, whereas 44 (23.1%) of 191 African Brazilians had α-thalassemia: 41 (21.5%) were heterozygous (-α^3.7^/αα) and 3 (1.6%) were homozygous (-α^3.7^/-α^3.7^) for the -α^3.7^ deletion genotype. Among 101 subjects with mild anemia and microcytosis, 32 (31.7%) had α-thalassemia, of which 23 (22.8%) were heterozygous for the -α^3.7^ deletion and 9 (8.9%) were homozygous (-α^3.7^/-α^3.7^). The -α^3.7^ allele frequency observed was 0.02, 0.12 and 0.20 for Euro and African Brazilians and patients with microcytic anemia, respectively. The frequency of α-thalassemia in patients with microcytic anemia was significantly higher than in healthy volunteers, regardless of their ethnic origin (p = 0.001).

The blood parameters in individuals with α-thalassemia genotypes, β-thalassemia trait and volunteers are shown in Table 2. All of the volunteers had an MCV of 81.2-103.6 fL. MANOVA revealed significant differences in the hematological parameters of the various groups (-α^3.7^/αα, -α^3.7^/-α^3.7^, β-thalassemia trait and volunteers) (Pillai's trace for comparison between genotypes: F = 38,58; p < 0.001). Subsequent univariate analyses of the values for Hb, Hct and MCHC clearly separated the volunteers from patients, but there was no difference among the various groups of patients. On the other hand, MCV and MCH discriminated among the four groups of individuals and there was a trend from high values in volunteers to low values in β-thalassemia trait patients, with intermediate values for -α^3.7^/αα and -α^3.7^/-α^3.7^. Healthy volunteers and -α^3.7^/αα subjects had similar RBC numbers (4.6 x 10^12^/L) that were significantly lower than in β-thalassemia trait carriers; individuals with -α^3.7^/-α^3.7^ had an intermediate number of RBCs that was not significantly different from the other groups.

Microcytic hypochromic anemia is a common hematological condition in clinical practice. As shown here, α-thalassemia, represented by the -α^3.7^ deletion, is a common cause of this hematological alteration (present in 31.7% of microcytic patients). These results agree with those reported by Borges *et al.* (2001) for a southeastern Brazilian population in which α-thalassemia explained about 50% of the cases with microcytosis. Other studies that have used an approach similar to that described here have reported α-thalassemia in 25%-80% of microcytosis patients in European or European-derived populations (Foglietta *et al.*, 1996; Sivera *et al.*, 1997; Bergeron *et al.*, 2005; Di Bella *et al.*, 2006; Lafferty *et al.*, 2007).

This is the first report of the prevalence of α-thalassemia in the general Brazilian population of European ancestry. The -α^3.7^ allele frequency observed here was 0.02, which is similar to that observed in Portuguese (3.5%) (Peres *et al.*, 1995) and Italians (5%) (Velati *et al.*, 1986) with whom southern Brazilians of European descent share common ancestors, but was significantly (p = 0.001) lower than in African Brazilians (Table 1). In the latter group, α-thalassemia genotypes were present in 23.1% of the population, with an allele frequency of 0.12. As expected in admixed populations such as the African Brazilians investigated here, this allele frequency was somewhat lower than in African populations, where the frequency of -α^3.7^ α-thalassemia ranges from 16.8% in Guinea-Bissau (Masmas *et al.*, 2006) to 24% in Senegal (Migot-Nabias *et al.*, 2006). The frequency of the -α^3.7^ allele in this study was similar to that of three out of four Brazilian populations of African ancestry investigated by others (Sonati *et al.*, 1991; Adorno *et al.*, 2005), despite different degrees of African admixture, and much higher than the prevalence described in northern Brazil (7%) where Amerindian admixture is higher than African admixture (Souza *et al.*, 2009).

Among patients with mild anemia and microcytosis that remained undiagnosed, it is possible that the non-deletional forms of α-thalassemia determined by the -α^Hph^ and -α^NcoI^ alleles, which are common in Mediterranean populations (Foglietta *et al.*, 1996; Kattamis *et al.*, 1996; Di Bella *et al.*, 2006), could explain a proportion of these cases, mainly among those of European ancestry. In addition, rare deletional forms not included in the PCR multiplex reaction could also be present in some patients.

As shown in Table 2, the values for various blood parameters were significantly different among healthy volunteers and individuals with α-thalassemia genotypes and the β-thalassemia trait. In contrast to ß -thalassemia carriers, for which important regional differences in the mutational profile have been identified in Brazilian populations (Reichert *et al.*, 2008), α-thalassemias show considerably less variability, which greatly facilitates the implantation of adequate public health policies and diagnostic services for dealing with this frequent genetic trait and for diagnosing microcytosis. In this context, the data reported here may serve as potential reference values for the detection of Brazilian patients suspected of having thalassemia. Finally, in individuals with microcytosis that is unrelated to an iron deficiency or an inflammatory state, the identification of α-thalassemia can avoid the need for expensive investigations to define the etiology of the anemia and eliminate unnecessary iron supplementation.

## Figures and Tables

**Table 1:** α^3.7^ allele and genotype frequencies.

	Allelic frequencies			Genotypes		
Subjects	Αα	-α^3.7^		αα/αα	-α^3.7^/αα	-α^3.7^/-α^3.7^
Euro-descendants	0.98	0.02		192 (95.5)*	9 (4.5)	0
African Brazilians	0.88	0.12		147 (77.0)	41 (21.5)	3 (1.6)
p	< 0.001	< 0.001			< 0.001	
Patients with microcytic anemia	0.80	0.20		69 (68.3)	23 (22.8)	9 (8.9)
p^§^	< 0.001	< 0.001		< 0.001	< 0.001	0.027
p^#^	< 0.001	< 0.001		0.01		< 0.001

*Number of individuals and percentage (in parentheses). ^§,#^compared with Euro-descendants and African Brazilians, respectively.

**Table 2:** Blood indices in α-thalassemia genotypes, β-thalassemia trait and healthy individuals.

Blood index	Subjects				p
	-α^3.7^/αα	-α^3.7^/-α^3.7^	β-thalassemia	Healthy	
Hb (g/dL)*	11.5 ± 0.23^a^	11.3 ± 0.16^a^	11.2 ± 0.13^a^	14.1 ± 0.12^b^	< 0.001
RBC (x 10^12^/L)*	4.65 ± 0.12^a^	5.00 ± 0.17^a, b^	5.47 ± 0.06^b^	4.66 ± 0.04^a^	< 0.001
Hct (%)*	35.8 ± 0.70^a^	35.8 ± 0.58^a^	35.4 ± 0.39^a^	42.5 ± 0.33^b^	< 0.001
MCV (fL)	77.3 ± 1.35^a^	71.6 ± 1.77^b^	67.0 ± 1.44^c^	91.3 ± 0.38^d^	< 0.001
MCH (pg)	24.9 ± 0.47^a^	22.6 ± 0.73^b^	21.4 ± 0.57^c^	30.2 ± 0.14^d^	< 0.001
MCHC (%)	31.9 ± 0.23^a^	31.6 ± 0.35^a^	31.9 ± 0.38^a^	33.1 ± 0.76^b^	< 0.001

The values are the mean ± SEM. Hb: hemoglobin, RBC: red blood cell, Hct: hematocrit, MCV: mean corpuscular volume, MCH: mean corpuscular hemoglobin, and MCHC: mean corpuscular hemoglobin concentration. Means identified by the same letters did not differ significantly by the SNK test, except for RBC, for which the Tamhane test was used. *Analyzed using values corrected for age and sex.
